# Genome‐wide association studies of fruit quality traits in jujube germplasm collections using genotyping‐by‐sequencing

**DOI:** 10.1002/tpg2.20036

**Published:** 2020-08-07

**Authors:** Lu Hou, Wu Chen, Zhiyong Zhang, Xiaoming Pang, Yingyue Li

**Affiliations:** ^1^ Beijing Advanced Innovation Center for Tree Breeding by Molecular Design National Engineering Laboratory for Tree Breeding, College of Biological Sciences and Technology Beijing Forestry University Beijing 100083 China; ^2^ The Germplasm Bank of Wild Species Kunming Institute of Botany Chinese Academy of Sciences Kunming 650201 China; ^3^ Beijing Key Laboratory of Ornamental Plants Germplasm Innovation and Molecular Breeding National Engineering Research Center for Floriculture Beijing Laboratory of Urban and Rural Ecological Environment Key Laboratory of Genetics and Breeding in Forest Trees and Ornamental Plants of Ministry of Education, School of Landscape Architecture Beijing Forestry University Beijing 100083 China

## Abstract

Chinese jujube (*Ziziphus jujuba* Mill.) is an important fruit crop and harbors many highly diverse traits of potential economic importance. Fruit size, stone size, and fruit cracking have an important influence on the commercial value of jujube. This study is the first to conduct a genome‐wide association study (GWAS) on 180 accessions of jujube and focuses on locating single‐nucleotide polymorphisms (SNPs) associated with nine important fruit quality traits. Genotyping was performed using genotyping‐by‐sequencing and 4651 high‐quality SNPs were identified. A genetic diversity analysis revealed the presence of three distinct groups, and rapid linkage disequilibrium decay was observed in this jujube population. Using a mixed linear model, a total of 45 significant SNP–trait associations were detected, among which 33 SNPs had associations with fruit size‐related traits, nine were associated with stone size‐related traits, and three with fruit cracking‐related traits. In total, 21 candidate genes involved in cell expansion, abiotic stress responses, hormone signaling, and growth development were identified from the genome sequences of jujube. These results are useful as basic data for GWAS of other jujube traits, and these significant SNP loci and candidate genes should aid marker‐assisted breeding and genomic selection of improved jujube cultivars.

**Abbreviations**
CI: cracking index
CL: cracking level
CR: cracking rate
CV: coefficient of variation
FLD: fruit longitudinal diameter
FSI: fruit shape index
FTD: fruit transverse diameter
FW: fruit weight
GBS: genotyping‐by‐sequencing
GLM: generalized linear model
GWAS: genome‐wide association study
K: kinship
LD: linkage disequilibrium
MLM: mixed linear model
NJ: neighbor‐joining
PCoA: principal coordinate analysis
Q: population structure
Q‐Q: quantile‐quantile
QTL: quantitative trait locus
SLD: stone longitudinal diameter
SNP: single nucleotide polymorphism
SSR: simple sequence repeat
STD: stone transverse diameter.

## 1 INTRODUCTION

Chinese jujube (*Ziziphus jujuba* Mill.) is an important member of the Rhamnaceae family originating in China with a documented domestication history of more than 7000 years (Liu & Cheng, 1995). Jujube fruit is nutritionally and medicinally valuable. The fruit is rich in protein, calcium, iron, magnesium, and ascorbic acid, and also contains numerous trace elements, all of which contribute to the commercial value of the species (Gao, Wu, & Wang, 2013). Fruit cracking is among the main problems that can affect jujube fruit. As a result, farmers can suffer significant economic losses because of the damage to their commodity (Khadivi‐Khub, 2015). This problematic fruit cracking is also commonly observed in *Vitis vinifera* (Jáuregui‐Riquelme, Kremer‐Morales, Alcalde, & Pérez‐Donoso, 2017), *Litchi chinensis* (Marboh et al., 2017), *Malus domestica* (Kasai, Hayama, Kashimura, Kudo, & Osanai, 2008), *Prunus avium* (Balbontín et al., 2013), *Punica granatum* (Saei, Sharifani, Dehghani, Seifi, & Akbarpour, 2014), and *Solanum lycopersicum* (Yang et al., 2016). Plant organ size is an important agronomical trait targeted during domestication. In the case of fruit tree breeding, large fruit and small stones are the most desired features (Zhang, Fan, Zhang, Jiang, & Liu, 2017). Correlations are observed among fruit size, stone size, and fruit cracking of jujube (Chen et al., 2017a). These relationships make it challenging to enhance one sought‐after trait without compromising another trait. Understanding the genes and genomic regions that control these important traits is essential in order to improve the genetic composition of jujube.

Several previous studies have determined that many pomological traits, including fruit size, stone size, and fruit cracking related to fruit quality and productivity, are quantitative traits controlled by multiple genes (Iwata, Minamikawa, Kajiya‐Kanegae, Ishimori, & Hayashi, 2016; Qi et al., 2015). For instance, Quilot et al. (2004) identified three main genomic regions where alleles from *Prunus davidiana* produced adverse effects on multiple traits, including stone and fruit sizes. Recently, 54 quantitative trait loci (QTLs) associated with several traits, including stone weight (G1 and G7), in *Musa* spp. were identified (Sardos et al., 2016). Six QTLs were detected on four of the eight linkage groups for *P. avium* fruit size, and a QTL for cracking susceptibility on LG5 was detected (Quero‐Garcia et al., 2012; Rosyara et al., 2013). Two QTLs, *Ck3* and *Ck10* on chromosome 3 and chromosome 10, respectively, were identified in single‐ and multiple‐environment QTL analysis of the cracking trait in tomato (Capel et al., 2017). Plant organ size is genetically determined by both cell division and cell expansion (Horiguchi, Ferjani, Fujikura, & Tsukaya, 2006; Li & Li, 2016). Several studies have suggested that fruit and stone size are controlled by multiple factors, including plant hormones, microRNAs, and cytochrome P450s (Nitsch et al., 2012; Qi, Liu, Song, Li, & Li, 2017; Yao et al., 2015). The FW 2.2 (*fw2.2*) and cell number regulator (*CNR*) genes are known to be primarily responsible for regulating fruit size (Guo & Simmons, 2011). Stone development is affected by physiological and genetic factors, which lead to embryo abortion and degradation and, ultimately, to stone degradation (Tang et al., 2018). Peel morphology, physio‐biochemical characteristics, and fruit development influence fruit cracking. Recent studies have revealed a close relationship between phytohormones and fruit cracking (Li et al., 2014). Polygalacturonase (*SlPG*) and expansin (*SlEXP1*) proteins cooperatively disassemble the tomato fruit cell walls during ripening and thereby influence tomato cracking (Jiang et al., 2019). Compared with other fruit trees, little is known about the genetic factors responsible for important fruit traits in jujube.

**Core Ideas**

- Considerable variation was observed for nine fruit quality traits in jujube.
- Population structure and linkage disequilibrium were studied with GBS‐derived markers.
- GWAS detected significant trait associations using a MLM model.
- Candidate genes for fruit size, stone size, and fruit cracking were identified.

A number of recent advances in genomic technologies have been used to investigate the genetic basis of variation in large germplasm sets by genome‐wide association studies (GWAS). In recent years, GWAS has been a popular method used for identifying the associated markers or causal genes responsible for important traits (Rosenberg et al., 2010). GWAS have outperformed bi‐parental QTL mapping because the former do not require the development of segregating populations. GWAS exploit the recombination events present in a large number of unrelated individuals, thus providing higher mapping resolution (Burghardt, Young, & Tiffin, 2017; Huang & Han, 2014). In the GWAS approach, the collections of unrelated genotypes exhibit much more limited linkage disequilibrium (LD) between pairs of neighboring markers, and therefore a large number of markers are needed to cover the genome (Sonah, O'Donoughue, Cober, Rajcan, & Belzile, 2015). Genotyping‐by‐sequencing (GBS), a complexity reduction approach based on restriction‐enzyme digestion, can efficiently generate a large number of single nucleotide polymorphisms (SNPs) distributed throughout the genome for conducting an effective GWAS (He et al., 2014; Lee et al., 2017). GWAS based on GBS‐derived markers has been used to study population structure and genetic relationships, and identify novel genes influencing crucial traits in rice (Begum et al., 2015), wheat (Juliana et al., 2013), maize (Wallace et al., 2014), sorghum (Morris et al., 2013), and soybean (Sonah et al., 2015). Many candidate genes have been identified based on the estimated phenotypic variation and nucleotide polymorphisms, but it is challenging to further characterize the unknown genes of species with complicated genomes. In contrast to crop species, it is difficult to study the phenotype of fruit trees by GWAS. This is because of breeding difficulties that occur as a result of the long juvenile stage, the large physical stature of the plants, and the large genome. A recent study detected SNPs significantly associated with skin chroma and acidity on chromosomes 3 and 9 of Korean apple germplasm collections by GBS‐GWAS (Lee et al., 2017). In recent years, whole‐genome assemblies of Chinese jujube cultivars ‘Dongzao’ and ‘Junzao’ were released (Huang et al., 2016; Liu et al., 2014), which enables researchers to identify candidate genes if the trait mapped intervals can be sufficiently resolved.

In this study, GWAS was successfully used to analyze nine important fruit quality traits by examining natural variation in 180 jujube accessions and using GBS technology. The goal of this research was to carefully examine population structure, identify the genetic underpinnings regulating fruit quality traits, and enhance marker‐assisted breeding of jujube. We also observed the peak GWAS signals and candidate genes for the first time in jujube. These findings will be useful as basic data for GWAS on other jujube populations and traits.

## 2 MATERIALS AND METHODS

### 2.1 Plant materials

This study included 180 Chinese jujube accessions (150 accessions in the core collection previously described by Xu et al. (2016), 15 extreme cracking‐resistant accessions, and 15 extreme cracking‐susceptible accessions). Leaf samples and phenotypic data for these accessions were collected with permission from two locations: the National Foundation for Improved Cultivar of Chinese Jujube, Cangzhou County, Hebei Province, China (Cangzhou), and the National Chinese Jujube Germplasm Repository located in Taigu County, Shanxi Province, China (Taigu). The complete list of accessions used in this study is reported in Supplemental Table S1. All leaves collected for each accession were rapidly frozen in liquid nitrogen and stored at −80°C until used.

### 2.2 Phenotyping

Nine fruit quality traits, namely fruit transverse diameter (FTD), fruit longitudinal diameter (FLD), fruit weight (FW), fruit shape index (FSI), stone transverse diameter (STD), stone longitudinal diameter (SLD), cracking rate (CR), cracking level (CL), and cracking index (CI), in the 180 Chinese jujube accessions were measured for three continuous years from 2014–2016 in two locations. A total of 30 half‐red fruits (uniform intact jujube fruit) of each accession were collected, with ten individuals per replicate. Jujube fruit cracking was induced by the natural immersion method, and the CR, CL and CI were measured after 48 h, as described by Chen et al. (2017a). The FTD and FLD (maximum value) was measured with a vernier caliper, respectively. A knife was used to separate the fruit from the stone, the stone was washed, and then STD and SLD was measured. Phenotypic data were unavailable for some accessions because of the absence of a fruiting tree or lack of fruits. The average value for each trait from three replicates for three years for each accession was used for statistical analysis and GWAS analysis. Phenotypic data for the nine fruit traits are shown in Supplemental Table S2 and were analyzed using Microsoft EXCEL 2013 and SPSS 19.0 software. Broad‐sense heritability (*H* ^2^) per location was computed by analysis of variance (ANOVA) using the R software package “lme4” (Bates, Mächler, Bolker, & Walker, 2015; Singh, Ceccarelli, & Hamblin, 1993). Correlation analysis for all traits was conducted using the R software package “corrplot”.

### 2.3 DNA preparation, GBS library preparation, and sequencing

Total genomic DNA from the 180 accessions was extracted from fresh leaves using a plant genomic DNA kit (Biomed Gene Technology Co., Ltd, Beijing, China). The integrity, concentration, and purity of DNA were analyzed using 1% agarose gel electrophoresis and a Qubit 3.0 Fluorometer (Invitrogen, Carlsbad, CA, USA). For each GBS library construction, about 100 ng genomic DNA was digested with the *Ape*KI restriction enzyme. The common barcode adapters were ligated to the products of the restriction reaction after digestion, followed by PCR amplification, as described by Chen, Hou, Zhang, Pang, and Li (2017b). An Illumina HiSeq 4000 platform was used for 100‐bp pair‐end sequencing of the GBS libraries. Raw sequencing reads were demultiplexed according to the barcodes and the adapter, and barcode sequences were trimmed using a custom C script. Reads that could be mapped to multiple locations or included more than 50% low‐quality bases (quality value ≤ 5) were discarded. The clean reads were then aligned to the jujube reference genome version 1.1 (Liu et al., 2014) using BWA version 0.7.10 (Li & Durbin, 2009) with default parameters. Variant calling was conducted using GATK version 3.2.2 (McKenna et al., 2010).

### 2.4 Population genetic analysis

To evaluate the population genetic structure, STRUCTURE version 2.3.3 based on a Bayesian clustering analysis was run with the number of populations (*K*) set from one to seven (Pritchard, Stephens, & Donnelly, 2000). For each *K*, 20 independent runs were completed with a burn‐in period of 100,000 iterations and a Markov chain Monte Carlo of 100,000 iterations. We determined the optimal *K* value by calculating Δ*K* using STRUCTURE HARVESTER (Earl & Vonholdt, 2012). Genetic distances between pairs of accessions were calculated, and a principal coordinate analysis (PCoA) was performed using GenAlEx version 6.5 (Peakall & Smouse, 2012). Nei's genetic distance between individuals was calculated using PowerMarker version 3.51 with 1000 bootstrap replicates (Liu & Muse, 2004). An unrooted phylogenetic tree was constructed with the neighbor‐joining (NJ) algorithm using MEGA version 6.0 with 1000 bootstrap replicates based on the genetic distance matrix (Tamura, Stecher, Peterson, Filipski, & Kumar, 2013).

### 2.5 Linkage disequilibrium and genome‐wide association analysis

Population structure (Q) and kinship (K) were considered as a means to increase statistical power and minimize false positives. A generalized linear model (GLM) including the Q matrix, and a mixed linear model (MLM) including the Q and K matrices (Q + K matrices), were used to test marker–trait associations using TASSEL 5.0 software (Bradbury et al., 2007). On the basis of the quantile–quantile (Q‐Q) plot of *P*‐values, the uniform distribution of expected and observed −log_10_ (*P*‐value) for the different traits was compared. The significant association signals between the genomic SNP locus and all traits had the lowest *P*‐values and highest *R* ^2^ in Chinese jujube. The pairwise LD between the genome‐wide markers on each chromosome was estimated with their allele frequency correlations (*r* ^2^) using TASSEL 5.0 software (Bradbury et al., 2007). A LD decay plot was generated using the R software package “ggplot2”. The Q‐Q plot for assessment of the normal distribution of data and Manhattan plots were generated using the R software package “qqman”.

### 2.6 Putative candidate genes identification

Candidate genes that were located within the LD decay distance upstream and downstream of significant associated SNPs were identified in accordance with the method of Li, Wang, Ai, Li, and Song (2018). The physical positions of significant SNPs and gene annotations were obtained from the jujube reference genome version 1.1 (Liu et al., 2014).

## 3 RESULTS

### 3.1 Distribution and correlation of different traits

**FIGURE 1:**
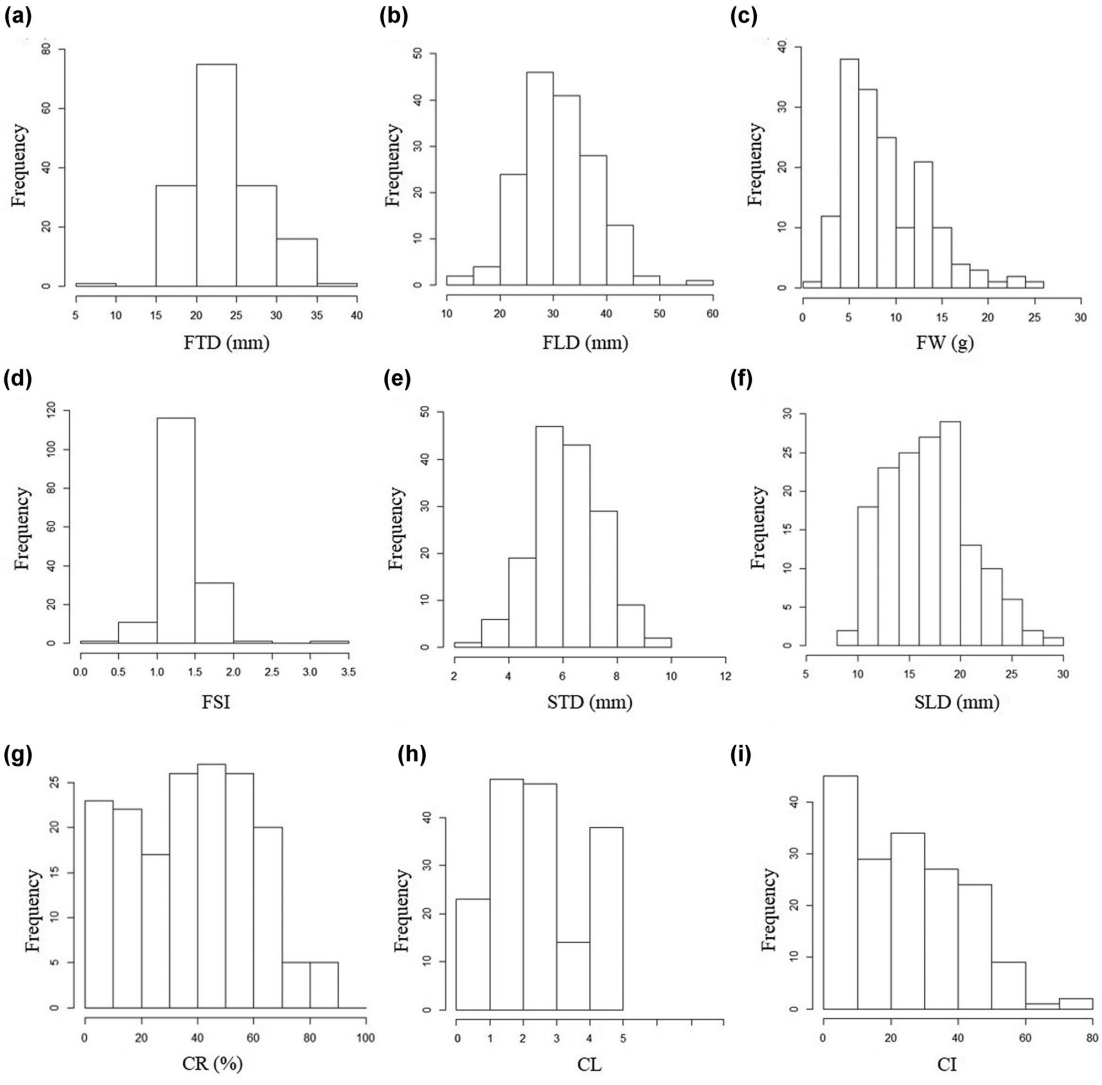
Frequency distribution of (a) fruit transverse diameter (FTD), (b) fruit longitudinal diameter (FLD), (c) fruit weight (FW), (d) fruit shape index (FSI), (e) stone transverse diameter (STD), (f) stone longitudinal diameter (SLD), (g) cracking rate (CR), (h) cracking level (CL) and (i) cracking index (CI) in 180 jujube accessions

Phenotyping was conducted for three years to study the distribution and correlation of nine fruit quality traits. All phenotypic traits except for cracking traits were approximately normally distributed and no traits showed a multi‐modal pattern or clearly separated into two or more classes (Figure 1). The traits CR, CL, and CI exhibited distinct continuous distribution trends, which indicated the existence of different grades of cracked fruit materials in the core collection. These results verified that these characteristics of jujube fruit are quantitative traits.

**FIGURE 2:**
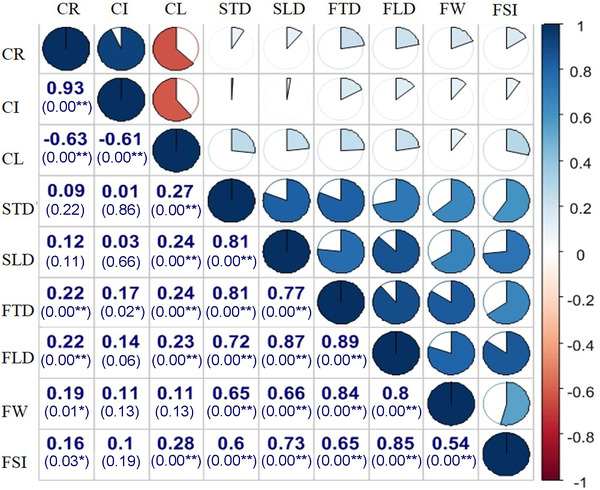
Phenotypic correlations among nine fruit traits. The pie symbols represent the strength of correlation coefficient, red represents negative correlation and blue represents positive correlation. *P*‐value for significant correlations is shown at the bottom. ^*^Significant at the .05 probability level. ^**^Significant at the .01 probability level. FTD, fruit transverse diameter; FLD, fruit longitudinal diameter; FW, fruit weight; FSI, fruit shape index; STD, stone transverse diameter; SLD, stone longitudinal diameter; CR, cracking rate; CL, cracking level; CI, cracking index

A broad range of coefficient of variation (CV) values was observed, with the lowest CV (19.47%) observed for FTD. The CI ranged from 0 to 0.91 and showed the highest CV (94.91%) (Table 1). The ANOVA for the nine fruit traits revealed a significant effect of genotype (*P* < 0.001). High *H* ^2^ was observed for all traits, with CL at Taigu being the only exception (*H* ^2^ = 0.68), which reflected a considerable genetic component underlying the accession variation (Table 1). All correlations between traits were positive, except for those between CL and CR (*r* = −0.63), and CL and CI (*r* = −0.61). In particular, a strong positive correlation was observed between CR and CI (*r* = 0.93); this relationship was attributable to the observation that high CR, a consequence of high susceptibility to cracking, generally increases the CI (Figure 2).

**TABLE 1:** Descriptive statistics and diversity test of nine fruit traits

				Cangzhou				Taigu			
Trait	Mean	SD	CV (%)	G	E	G × E	*H* ^2^	G	E	G × E	*H* ^2^
FTD (mm)	23.46	4.57	19.47	^***^	^***^	^***^	0.95	^***^	^***^	^***^	0.88
FLD (mm)	30.83	7.07	22.78	^***^	^***^	^***^	0.96	^***^	^***^	^***^	0.96
FW (g)	8.92	4.53	50.79	^***^	^***^	^***^	0.95	^***^	^***^	^***^	0.90
FSI	1.34	0.28	21.22	^***^	^***^	^***^	0.96	^***^	^***^	^***^	0.98
STD (mm)	6.16	1.23	19.93	^***^	ns^†^	^***^	0.88	^***^	ns	^***^	0.92
SLD (mm)	16.93	4.04	23.88	^***^	^***^	^***^	0.95	^***^	^***^	^***^	0.98
CR (%)	40.98	0.30	72.07	^***^	ns	^***^	0.78	^***^	ns	^***^	0.74
CL	2.96	1.35	45.53	^***^	ns	^***^	0.75	^***^	ns	^***^	0.68
CI	0.22	0.21	94.91	^***^	^***^	^***^	0.77	^***^	^***^	^***^	0.85

FTD, fruit transverse diameter; FLD, fruit longitudinal diameter; FW, fruit weight; FSI, fruit shape index; STD, stone transverse diameter; SLD, stone longitudinal diameter; CR, cracking rate; CL, cracking level; CI, cracking index; SD, standard deviation; CV, coefficient of variation; G × E, genotype‐by‐environment interaction; *H* ^2^, broad‐sense heritability.

^***^Significant at the .001 probability level. ^†^ns, nonsignificant.

### 3.2 Population structure and linkage disequilibrium

**FIGURE 3:**
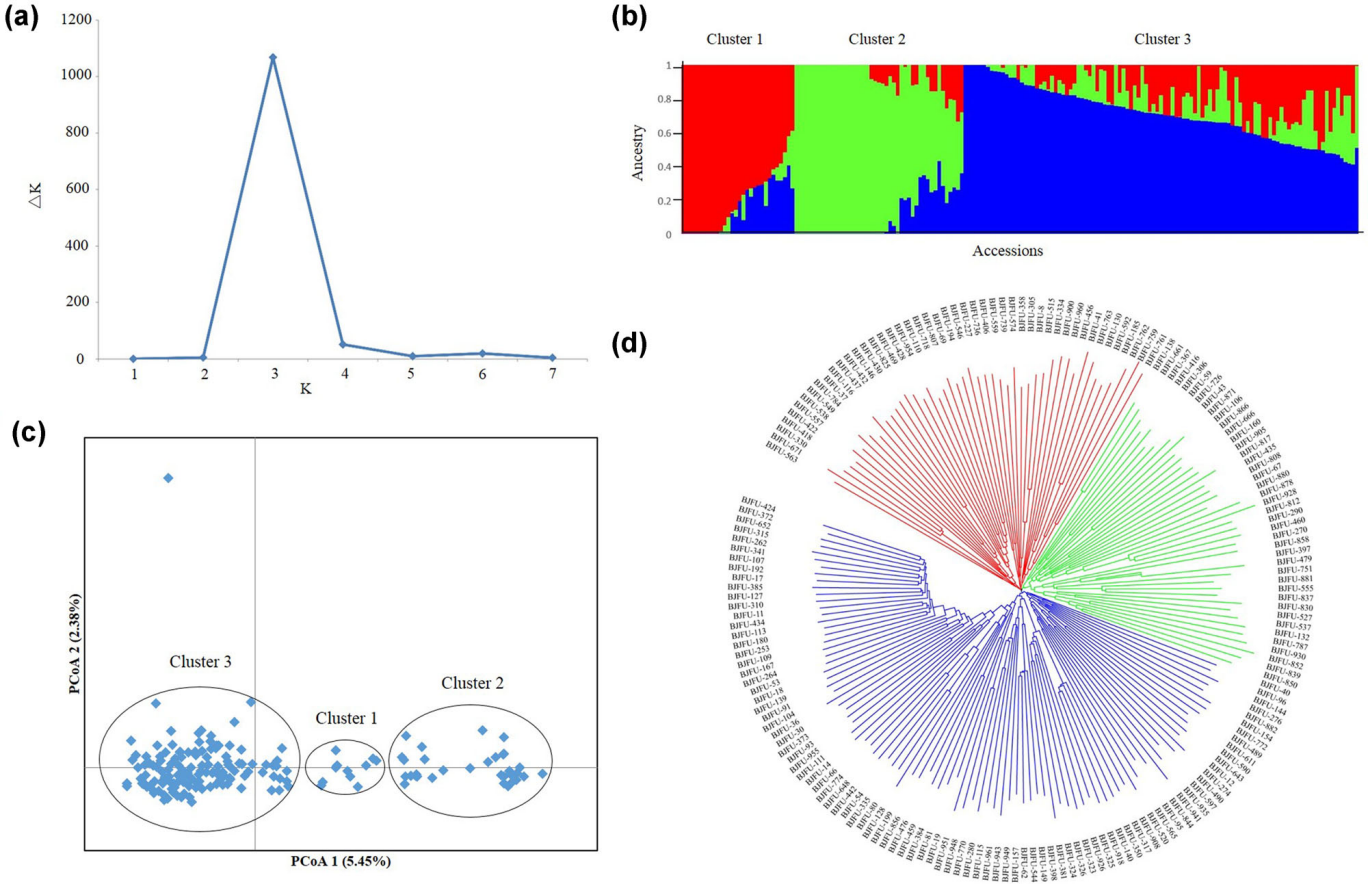
Population structure of 180 jujube accessions based on single nucleotide polymorphism (SNP) makers. (a) Estimation of population using LnP(D)‐derived △*K* with *K* ranged from 1 to 7. (b) Population structure based on *K* = 3. The x‐axis is the jujube accessions, and the length of the colored bar (y‐axis) shows the accessions’ estimated proportion of membership to each cluster. (c) Principal coordinate analysis (PCoA) plots of first two components. PCoA 1 and PCoA 2 indicate score of principal components 1 and 2, respectively. Values in parentheses indicate percentage of variance in the data explained by each principal component. (d) Phylogenetic tree constructed using neighbor‐joining (NJ) method. Each color represents a cluster

Sequencing of GBS libraries constructed from the 180 jujube accessions produced 173.331 Gb of clean reads (data not shown). After removal of SNPs with missing data >20% and minor allele frequency ≤0.05, 4651 high‐quality SNPs were retained and 3986 (85.7%) were physically mapped across the 12 jujube chromosomes (Supplemental Table S3). Based on these SNPs, all pairwise genetic distances among the 180 jujube accessions were assessed. Population structure based on the entire panel was assessed for values of *K* ranging from 1 to 7. The optimal number of genetic clusters determined using STRUCTURE HARVESTER was 3 as Δ*K* was largest at *K* = 3 (Figure 3a). Cluster 3 contained the highest number of accessions (105, with 47.62% from Cangzhou and 52.38% from Taigu), followed by Cluster 2 (45, with 84.44% from Cangzhou and 15.56% from Taigu) and Cluster 1 (30, with 53.33% from Cangzhou and 46.67% from Taigu) (Figure 3b). The PCoA analysis indicated that diversity was present among the jujube genotypes. The first two PCoA components explained 5.45% and 2.38% of the total variation, respectively (Figure 3c). More than 50% of the jujube accessions were assigned to Cluster 3. Cluster 2 mostly contained accessions collected from Cangzhou, which were much more scattered than those in Clusters 1 and 3. A NJ tree constructed from a matrix of genetic distances also resolved the individuals into three divergent clusters. These three clusters (labeled in green, blue, and red, respectively, in Figure 3d) contained 41, 92, and 47 accessions, respectively.

**FIGURE 4:**
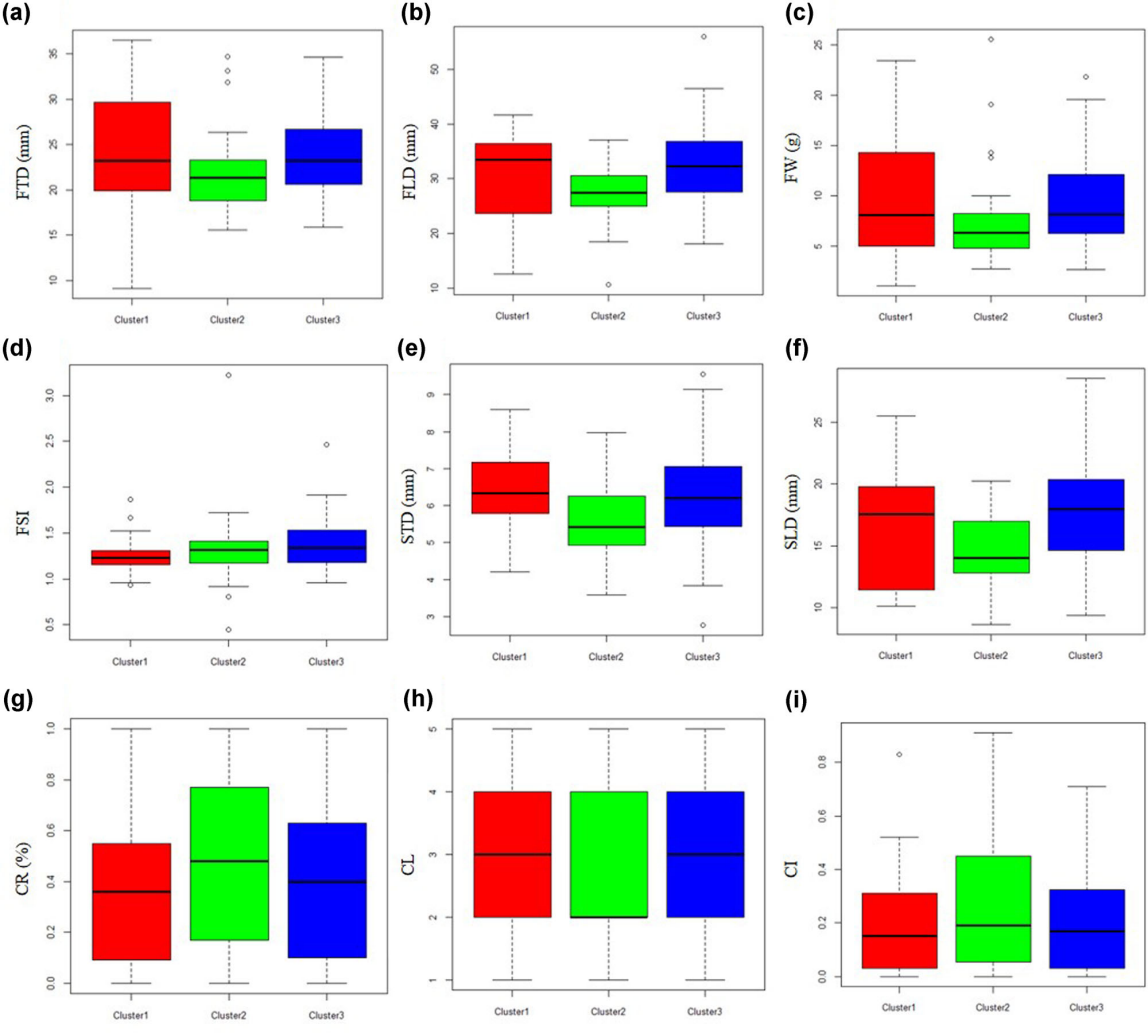
Boxplots showing mean, median and range of phenotypic variation for the three jujube clusters for (a) fruit transverse diameter (FTD), (b) fruit longitudinal diameter (FLD), (c) fruit weight (FW), (d) fruit shape index (FSI), (e) stone transverse diameter (STD), (f) stone longitudinal diameter (SLD), (g) cracking rate (CR), (h) cracking level (CL) and (i) cracking index (CI)

The nine fruit quality traits differed significantly among the three clusters resolved in the aforementioned analyses (Figure 4; Supplemental Table S4). Cluster 2 showed the smallest mean FTD, FLD, FW, STD, and SLD and the largest CR and CI, which indicated that fruit produced by accessions in Cluster 2 were, on average, smaller, lighter, and more susceptible to cracking. The highest CV among all three clusters was observed for CI.

The genome‐wide LD decay, corresponding to the distance over which *r* ^2^ decreased from an initial value of 0.512 to 0.1, was approximately 10 kb (Supplemental Figure S1; Supplemental Table S5). The distribution of *r* ^2^ values with respect to physical distance is presented for each chromosome in Supplemental Figure S2. Within the collection of accessions, a rapid decay in LD was observed with increasing physical distance. The LD decay varied across different jujube chromosomes, with the slowest LD decay observed for chromosome 10 (50 kb) (Supplemental Table S6).

### 3.3 Marker–trait associations for fruit quality traits

We applied GLM (Q matrix) and MLM (Q + K matrices) models to analyze the associations between the nine fruit traits and the 4651 SNPs evaluated for the 180 Chinese jujube accessions (Yu et al., 2006). On the basis of the Q‐Q plots, the models performed reasonably well in controlling false positive associations potentially resulting from the underlying population structure. Compared with the GLM model, the MLM model was able to effectively control subpopulation structure and detect the most significant SNP markers for a given trait (Supplemental Figure S3; Supplemental Figure S4). Thus, the MLM model was used to test marker–trait associations.

**FIGURE 5:**
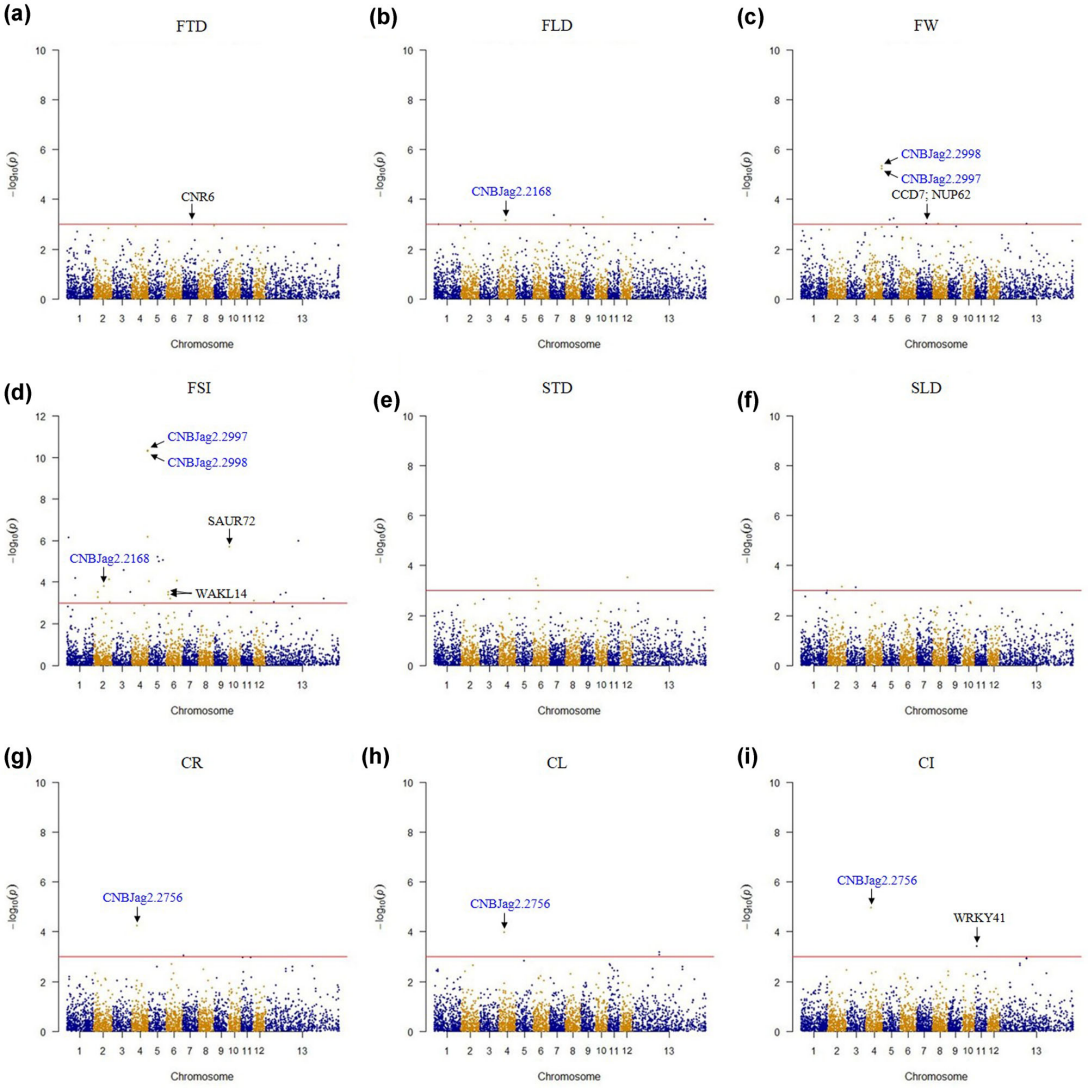
Manhattan plots of marker‐trait association for (a) fruit transverse diameter (FTD), (b) fruit longitudinal diameter (FLD), (c) fruit weight (FW), (d) fruit shape index (FSI), (e) stone transverse diameter (STD), (f) stone longitudinal diameter (SLD), (g) cracking rate (CR), (h) cracking level (CL) and (i) cracking index (CI) using mixed linear model (MLM). The red line denotes the genome‐wide significance threshold. Putative candidate genes linked to significant markers are labeled and significant markers identified in multiple traits are highlighted by blue color

The GWAS conducted using the MLM model identified 45 SNPs, distributed across all jujube chromosomes except for chromosome 9, as significantly associated with the nine fruit quality traits (Figure 5). Marker *R* ^2^ values ranged from 0.0680 to 0.3141, with *P*‐values varying from 9.98 × 10^−4^ to 4.52 × 10^−11^. Among the identified SNPs, one, four, six, five, and four were significantly associated with FTD, FLD, FW, STD, and SLD, respectively. A higher number of significant markers was associated with FSI than with the other traits, namely 25 SNPs located across eight chromosomes. The significant SNPs on chromosome 1 were only associated with FSI, and these three SNPs were detected in a 10.5 Mb (2.9–13.4 Mb) region. The highest *R* ^2^ value, and the most strongly significant, was that for marker CNBJag2.2997 located on chromosome 4. With regard to fruit cracking, two, one, and two SNPs were significantly associated with CR, CL, and CI, respectively. One significant SNP each on chromosomes 11 and 8 was only associated with CI and FW, respectively (Table 2).

**TABLE 2:** Details of most significant marker loci associated with nine fruit traits identified by genome‐wide association study and based on jujube genome annotations

Trait	Chr.no.	Marker	Position	*P*‐value	*R* ^2^	Putative candidate genes
FTD	7	CNBJag2.3931	15694707	9.98×10^−4^	0.0905	CNR6
FLD	2	CNBJag2.2168	16501852	7.79×10^−4^	0.0845	uncharacterized LOC107411425
	4	CNBJag2.2791	10941375	6.75×10^−4^	0.0680	
	7	CNBJag2.3789	6264083	4.29×10^−4^	0.0752	
	10	CNBJag2.723	11949770	4.97×10^−4^	0.1039	
FW	4	CNBJag2.2997	25553990	5.90×10^−6^	0.1458	PUB4
	4	CNBJag2.2998	25553994	4.60×10^−6^	0.1490	PUB4
	5	CNBJag2.3147	11156749	6.55×10^−4^	0.0872	NFD4
	5	CNBJag2.3239	17354423	5.71×10^−4^	0.0711	
	7	CNBJag2.3930	15674658	9.59×10^−4^	0.0917	CCD7; NUP62
	8	CNBJag2.4189	9424055	9.66×10^−4^	0.0707	CYP81D1
FSI	1	CNBJag2.52	2910215	7.12×10^−7^	0.1741	VQ17
	1	CNBJag2.205	13435211	4.30×10^−4^	0.1158	
	1	CNBJag2.207	13435377	6.33×10^−5^	0.1167	
	2	CNBJag2.2034	6897179	5.15×10^−4^	0.0901	STP8
	2	CNBJag2.2049	7599138	2.88×10^−4^	0.0972	
	2	CNBJag2.2168	16501852	1.48×10^−4^	0.1051	uncharacterized LOC107411425
	2	CNBJag2.2268	25197767	7.41×10^−5^	0.1146	
	2	CNBJag2.2280	26008576	9.05×10^−4^	0.0826	
	3	CNBJag2.2540	18110684	2.64×10^−5^	0.1271	HMGB7
	3	CNBJag2.2619	28743430	2.89×10^−4^	0.1036	
	4	CNBJag2.2996	25553988	6.66×10^−7^	0.1768	PUB4
	4	CNBJag2.2997	25553990	4.52×10^−11^	0.3141	PUB4
	4	CNBJag2.2998	25553994	4.82×10^−11^	0.3131	PUB4
	4	CNBJag2.3039	27823050	8.89×10^−5^	0.1115	
	5	CNBJag2.3181	13651735	6.00×10^−6^	0.1508	GLR2.8
	5	CNBJag2.3225	15865698	1.01×10^−5^	0.1406	
	5	CNBJag2.3277	22562780	8.51×10^−6^	0.1454	
	6	CNBJag2.3376	4100156	3.78×10^−4^	0.0780	WAKL14
	6	CNBJag2.3378	4100158	2.90×10^−4^	0.0796	WAKL14
	6	CNBJag2.3417	7105213	5.97×10^−4^	0.0888	LA1; MYB3R1
	6	CNBJag2.3553	17796154	8.50×10^−5^	0.1248	
	10	CNBJag2.590	1435417	2.00×10^−6^	0.1722	SAUR72
	10	CNBJag2.611	3011483	9.22×10^−4^	0.0894	
	10	CNBJag2.745	14308603	3.42×10^−4^	0.0946	
	12	CNBJag2.1066	319701	7.32×10^−4^	0.0852	CAMBP25
STD	6	CNBJag2.3392	4894422	3.39×10^−4^	0.0759	DSE1
	6	CNBJag2.3435	8342343	6.21×10^−4^	0.0864	
	12	CNBJag2.1173	10278956	2.93×10^−4^	0.0971	CALS8; GLY3
	12	CNBJag2.1174	10278957	2.93×10^−4^	0.0971	CALS8; GLY3
	12	CNBJag2.1175	10278968	2.93×10^−4^	0.0971	CALS8; GLY3
SLD	2	CNBJag2.2238	23009481	6.74×10^−4^	0.0762	zinc finger BED domain‐containing protein RICESLEEPER 2
	2	CNBJag2.2239	23009491	6.74×10^−4^	0.0762	zinc finger BED domain‐containing protein RICESLEEPER 2
	2	CNBJag2.2240	23009498	6.74×10^−4^	0.0762	zinc finger BED domain‐containing protein RICESLEEPER 2
	3	CNBJag2.2514	14884230	7.27×10^−4^	0.0726	
CR	4	CNBJag2.2756	8369293	5.64×10^−5^	0.1188	PMAT1
	7	CNBJag2.3703	1669990	8.80×10^−4^	0.0774	
CL	4	CNBJag2.2756	8369293	1.04×10^−4^	0.1107	PMAT1
CI	11	CNBJag2.851	2149500	3.68×10^−4^	0.0831	WRKY41
	4	CNBJag2.2756	8369293	1.09×10^−5^	0.1402	PMAT1

FTD, fruit transverse diameter; FLD, fruit longitudinal diameter; FW, fruit weight; FSI, fruit shape index; STD, stone transverse diameter; SLD, stone longitudinal diameter; CR, cracking rate; CL, cracking level; CI, cracking index.

Four markers were observed to be significantly associated with more than one trait. Marker CNBJag2.2168 was significantly associated with FLD and FSI, which were two highly correlated traits in the studied population. Two significant markers, CNBJag2.2997 and CNBJag2.2998, were both detected for FW and FSI. Marker CNBJag2.2756 located on chromosome 4 was significantly associated with three fruit cracking‐related traits. The remaining markers were associated with only one trait. In addition, some chromosomal regions were determined to be associated with multiple fruit traits. For example, a 3.0 Mb (23.0–26.0 Mb) interval on chromosome 2 was revealed to show associations with FSI and SLD, whereas a 4.2 Mb (4.1–8.3 Mb) interval on chromosome 6 was associated with FSI and STD. Significant marker loci associated with FW and FSI were mapped to a 11.4 Mb (11.2–22.6 Mb) interval on chromosome 5. A 9.4 Mb (6.3–15.7 Mb) region on chromosome 7 harbored three significant markers, one each for FTD, FLD, and FW (Table 2).

### 3.4 Identification of candidate genes

We predicted 21 candidate genes located within 10 kb upstream or downstream of 25 significant markers in the jujube genome (Table 2). On chromosome 7, marker CNBJag2.3931 associated with FTD was linked to *CNR6*. For FW, five candidate genes, namely U‐box domain‐containing protein 14 (*PUB4*), nuclear fusion defective 4 (*NFD4*), carotenoid cleavage dioxygenase 7, chloroplastic (*CCD7*), nuclear pore complex protein NUP62 (*NUP62*), and cytochrome P450 81D1 (*CYP81D1*), were predicted for associated SNPs. For FSI, 10 candidate genes were identified showing homology to linked SNPs; among these genes, wall‐associated receptor kinase‐like 14 (*WAKL14*) was linked to markers CNBJag2.3376 and CNBJag2.3378 at the same locus. On chromosome 4, markers CNBJag2.2996, CNBJag2.2997, and CNBJag2.2998 at the same locus were linked to *PUB4*. For STD, three markers, namely CNBJag2.1173, CNBJag2.1174, and CNBJag2.1175 at the same locus on chromosome 12, were linked to putative callose synthase 8 (*CALS8*) and persulfide dioxygenase ETHE1 homolog, mitochondrial (*GLY3*). Marker CNBJag2.3392 located on chromosome 6 was linked to the gene decreased size exclusion limit 1 (*DSE1*). The zinc‐finger BED domain‐containing protein RICESLEEPER 2 was linked to three markers (CNBJag2.2238, CNBJag2.2239, and CNBJag2.2240) associated with SLD. For fruit cracking, markers on chromosomes 4 and 11 were predicted to be associated with phenolic glucoside malonyltransferase 1 (*PMAT1*) and probable WRKY transcription factor 41 (*WRKY41*), respectively.

## 4 DISCUSSION

### 4.1 Genetic diversity of the 180 jujube accessions

Given that population structure can cause spurious associations between markers and traits, association studies that do not account for it should be viewed with skepticism (Korte & Farlow, 2013; Matthies, van Hintum, Weise, & Röder, 2012). In a fruit GWAS that takes population structure into consideration, the proper selection of various germplasm resources for analysis is important. Jujube population structure has previously been studied using simple sequence repeat (SSR) (Xu et al., 2016) and SNP (Chen et al., 2017b) markers. Using 4651 GBS‐based SNPs, the present results were in agreement with our previous study in which 962 jujube accessions, including the 180 accessions in the present study, were classified into three groups on the basis of structural, PCoA, and NJ analyses (Xu et al., 2016). However, 150 jujube accessions were grouped into two clusters based on the △*K* value and the PCoA results. This discrepancy may be due to the number of populations (Chen et al., 2017b).

The LD decay against a known physical distance is an important parameter for determining the appropriate density and number of molecular markers for GWAS (Mather et al., 2007). In the present study of jujube, a cross‐pollinated species, the average extent of LD decay for the entire jujube genome was estimated to be 10 kb at *r* ^2^ < 0.1. In general, a faster LD decay rate and a smaller LD block results in higher mapping resolution (Zaidi et al., 2016). The LD values observed in the current study are similar to our previous findings for a core collection of 150 accessions in which *r* ^2^ declined from an initial value of 0.483 to 0.104 over 9.77 kb (Chen et al., 2017b), which is similar to results reported for sesame (Morris et al., 2013). Our results verify the accuracy of LD values in jujube and suggest that the present jujube population contained sufficient genetic diversity and an adequate number of accessions for GWAS.

### 4.2 Marker–trait associations

Despite the availability of a high‐quality, well annotated, complete genome sequence for jujube, GWAS are not yet commonplace for this species (Huang et al., 2016; Liu et al., 2014). Decoupling associations from population structure and kinship, which are regarded as the two main confounding factors, can minimize false‐positive signals in GWAS (Atwell et al., 2010; Riedelsheimer et al., 2012). Using the MLM (Q + K matrices) model, we conducted a GWAS of nine fruit quality traits for 180 jujube accessions based on GBS‐derived markers and thereby identified 45 significant SNPs located on 11 jujube chromosomes.

Stone size‐related traits were observed to be significantly associated with nine SNPs for STD and SLD. For fruit cracking‐related traits, three significant SNPs were detected on three chromosomes. Marker CNBJag2.2756 on chromosome 4 was associated with CR, CL, and CI, which indicated that loci associated with fruit cracking are closely distributed on chromosome 4 in jujube. The most strongly significant marker loci for FTD and FLD were mapped on chromosome 7, whereas the most highly significant marker loci for FW and FSI were mapped on chromosome 4. Some chromosomal regions harbored markers for multiple fruit size‐related traits; these included a 10.9–27.8 Mb interval on chromosome 4 associated with FLD, FW, and FSI, and a 6.3–15.7 Mb interval on chromosome 7 associated with FTD, FLD, and FW. Chromosomes 4 and 7 contained significant markers for the three traits, thus indicating that loci associated with fruit size traits were closely distributed on these two chromosomes. To the best of our knowledge, only a single previous study has mapped fruit size traits in jujube using an F_1_ population (Xu, 2012). A comparison of chromosomal regions could not be performed, however, as Xu (2012) study used SSR and AFLP markers and a discrepancy exists between linkage groups on the genetic map and jujube chromosome numbers (Arora et al., 2017). Upon further validation, the significant markers identified in the present study may be useful for marker‐assisted breeding to improve jujube fruit quality.

### 4.3 Putative candidate genes linked to marker loci for fruit quality traits

Although a number of genes that affect fruit size, stone size, and fruit cracking have been cloned in other plant species, an understanding of the genetic basis of these fruit traits remains limited in jujube. The whole‐genome sequence of jujube is a useful resource for identification of candidate genes that underlie marker loci associated with fruit quality traits in this species. In the present study, we identified 21 candidate genes from the genome sequences of jujube.

Several functional genes linked to the loci identified in the present study provide putative underlying candidate genes involved in determination of fruit quality. Genes controlling fruit size have been extensively studied in other fruit crops. For fruit size, marker CNBJag2.3931 associated with FTD was linked to *CNR6*, a gene that regulates cell number. *CNR* genes are putative orthologs of *fw2.2* in tomato, which governs a QTL accounting for 30% of fruit size variation (Guo et al., 2010). *CNR* genes of *Prunus* are associated with fruit size in sweet and sour cherries (De Franceschi et al., 2013). The FW‐associated SNP marker CNBJag2.3930 on chromosome 7 was observed to be linked to *CCD7*. *CCD7* is highly expressed in young kiwifruit and tomato fruits (Ledger, Janssen, Karunairetnam, Wang, & Snowden, 2010; Vogel et al., 2010). *CCD7* and *CCD8* are crucial enzymes in the biosynthesis of strigolactone and are associated with fruit and seed size in tomato (Kohlen et al., 2012; Vogel et al., 2010). The fruit size is predominantly determined by the number and volume of cells, which is the result of cell division and cell expansion during organogenesis. *WAKL14* was linked to markers CNBJag2.3376 and CNBJag2.3378 associated with FSI and is a member of the wall‐associated kinases (WAK) family of proteins, which are tightly associated with cell walls and play roles in cell expansion (Qiu et al., 2013; Verica & He, 2002). Plant hormones are known to modulate growth and development at various stages of the developing fruit. For FW and FSI, two highly correlated traits, *PUB4* was observed to be linked to markers CNBJag2.2997 and CNBJag2.2998 associated with both traits, which is a component of the complex cytokinin‐signaling networks that regulate a variety of plant processes (Wang, Wu, Yu, Yin, & Xia, 2017). In addition, *NUP62* associated with FW is a major negative regulator of auxin signaling (Boeglin et al., 2016), and the auxin‐responsive protein SAUR72 associated with FSI plays a role in the regulation of auxin transport (Qiu et al., 2013). Auxin and cytokinin regulate the division and expansion of cells, thereby promoting vegetative growth and increasing fruit size (Srivastava & Handa, 2005).

With respect to fruit cracking, the plant transcription factor *WRKY41* associated with CI performs a number of important functions, namely regulation of anthocyanin biosynthesis (Duan et al., 2018), hormone signaling pathways (Higashi et al., 2008), and osmotic stress responses (Chu et al., 2015). The hormone balance of the fruit plays an important role during fruit cracking (Khadivi‐Khub, 2015). Wang et al. (2019) reported that the pericarp of a cracking‐susceptible litchi cultivar shows higher abscisic acid, ethylene, and jasmonic acid contents, and lower auxin and brassinosteroid contents than those of a cracking‐resistant litchi cultivar. Cracking is the consequence of a critically high turgor pressure caused by osmotic water uptake and increase in fruit volume (Correia, Schoutenb, Silva, & Gonçalves, 2018). Considine and Kriedemann (2000) reported that cracking of grape berries occurs when the fruit are submerged in solutions of low osmotic potential.

Significantly, several candidate genes were associated with multiple traits. *PMAT1* was predicted for marker CNBJag2.2756 associated with CR, CL, and CI, which were three highly correlated fruit cracking traits. The underlying genes linked to marker CNBJag2.2168 associated with FLD and FSI remain uncharacterized. This is consistent with the high positive correlation between the two fruit size traits (*r* = 0.85). These candidate genes associated with two or more traits are also critical for fruit size and fruit cracking. Furthermore, the remaining candidate genes linked to marker loci identified in the present study were involved in plant growth and development. For instance, *MYB3R1* was predicted for marker CNBJag2.3417 associated with FSI and is a transcriptional repressor that regulates organ size (Kobayashi et al., 2015). The significant three markers (CNBJag2.2238, CNBJag2.2239, and CNBJag2.2240) on chromosome 2 associated with SLD were linked to the zinc‐finger BED domain‐containing protein RICESLEEPER 2, which may regulate global gene expression by recruiting other cellular factors to regulate plant growth and development (Knip, Pater, & Hooykaas, 2012). These candidate genes are also of interest, but further work is needed to validate their involvement in controlling the studied traits.

In summary, 45 genome‐wide significant SNPs responsible for nine fruit quality traits in jujube were identified in the present investigation using association mapping. To the best of our knowledge, this is the first GWAS to screen genetic loci associated with economically important traits in jujube. A set of traits associated with fruit size, stone size, and fruit cracking was observed to be polygenic. A total of 21 candidate genes involved in plant growth and development were identified, but their functions require further validation in model and non‐model species. Given that different mechanisms underlie diverse traits, loci associated with fruit size, stone size, and fruit cracking may operate via different pathways. Future studies that include gene expression and functional analysis are needed to identify the candidate genes. After validation, functional markers and genes can be used for marker‐assisted breeding of jujube cultivars with improved fruit quality and enhanced resistance to cracking.

## AUTHOR CONTRIBUTION STATEMENT

YL conceived and designed the experiments. LH and WC performed the field trial to collect phenotypic data. LH and WC analyzed all data. LH wrote the manuscript. ZZ and XP provided valuable suggestions on the manuscript. ZZ and YL revised the manuscript. YL obtained funding and is responsible for this article. All authors read and approved the manuscript.

## CONFLICT OF INTEREST

The authors declare that they have no competing interests.

## Supporting information

SUPPORTING MATERIAL

SUPPORTING MATERIAL

SUPPORTING MATERIAL

SUPPORTING MATERIAL

SUPPORTING MATERIAL

SUPPORTING MATERIAL

SUPPORTING MATERIAL

SUPPORTING MATERIAL

SUPPORTING MATERIAL

SUPPORTING MATERIAL
