# Supplementary material for: Genome‐wide association studies of fruit quality traits in jujube germplasm collections using genotyping‐by‐sequencing
Source: Plant Genome. 2020 Aug 7;13(3):e20036. doi: 10.1002/tpg2.20036 (PMC12807363; doi:10.1002/tpg2.20036)
Supplement: Supplementary file 5 [file TPG2-13-e20036-s005.docx]

**Supplemental Figure S2** Average linkage disequilibrium decay across 12 jujube chromosomes.
